# Next-generation sequencing with comprehensive bioinformatics analysis facilitates somatic mosaic *APC* gene mutation detection in patients with familial adenomatous polyposis

**DOI:** 10.1186/s12920-019-0553-0

**Published:** 2019-07-03

**Authors:** Borahm Kim, Dongju Won, Mi Jang, Hoguen Kim, Jong Rak Choi, Tae Il Kim, Seung-Tae Lee

**Affiliations:** 1 Department of Laboratory Medicine, Yonsei University College of Medicine, 50 Yonsei-ro, Seodaemun-gu, Seoul, 03722 Republic of Korea; 2 Department of Pathology, Yonsei University College of Medicine, Seoul, Korea; 3 Department of Internal Medicine and Institute of Gastroenterology, Brain Korea 21 PLUS Project for Medical Sciences Yonsei Cancer Prevention Center, Yonsei University College of Medicine, 50 Yonsei-ro, Seodaemun-gu, Seoul, 03722 Republic of Korea

**Keywords:** Next-generation sequencing, *APC*, Somatic mosaic mutation, Familial adenomatous polyposis, Colorectal cancer

## Abstract

**Background:**

Familial adenomatous polyposis (FAP) is an autosomal dominant colorectal tumor characterized by numerous adenomatous colonic polyps that often lead to colon cancer. Although most patients with FAP harbored germline mutations in *APC* gene, it was recently recognized that patients with clinical FAP, but without detectable pathogenic mutations, could be associated with somatic mosaic *APC* mutation.

**Methods:**

We reanalyzed the nest-generation sequencing (NGS) gene panel testing results of patients who were diagnosed with FAP, but did not have *APC* mutations, at Yonsei Cancer Prevention Center between July 2016 and March 2018. We tested several variant calling algorithms to identify low level mosaic variants. In one patient with a low frequency *APC* mutation, NGS analysis was performed together with endoscopic biopsy. Variant calling tools HaplotypeCaller, MuTect2, VarScan2, and Pindel were used. We also used 3′-Modified Oligonucleotides (MEMO)-PCR or conventional PCR for confirmation.

**Results:**

Among 28 patients with clinical suspicion of FAP but no detectable pathogenic variants of colonic polyposis associated genes, somatic mosaic pathogenic variants were identified in seven patients. The variant allele frequency ranged from 0.3 to 7.7%. These variants were mostly detected through variant caller MuTect2 and Pindel, and were further confirmed using mutant enrichment with MEMO-PCR.

**Conclusions:**

The NGS with an adequate combination of bioinformatics tools is effective to detect low level somatic variants in a single assay. Because mosaic *APC* mutations are more frequent than previously thought, the presence of mosaic mutations must be considered when analyzing genetic tests of patients with FAP.

**Electronic supplementary material:**

The online version of this article (10.1186/s12920-019-0553-0) contains supplementary material, which is available to authorized users.

## Background

Familial adenomatous polyposis (FAP, OMIM#175100) is an autosomal dominant colorectal tumor syndrome characterized by numerous adenomatous colonic polyps that are prone to progress to colon cancer. The majority of patients with FAP harbor a germline mutation in the *APC* gene on chromosome 5q21. A few other genes, such as *MUTYH, POLD1*, and *POLE*, are also associated with hereditary colonic polyposis [1–4]. However, one-fifth of patients with FAP are apparently sporadic without any familial history [5, 6]. It has been widely recognized that some of these sporadic FAP patients have somatic mosaic *APC* mutations [5–12].

Conventionally, genetic tests for hereditary cancer are performed with leukocyte DNA using PCR and Sanger sequencing. Unlike germline mutations, somatic mutations show various mutant allele frequencies in leukocytes. As a result, a small fraction of mosaic mutations are missed in routine genetic analyses optimized for germline variants, partly due to limited sensitivity of the testing method.

Next-generation sequencing (NGS) has been rapidly adopted in the clinical field. In combination with extensive bioinformatics analysis, NGS can identify a wide range of variants in a single assay, including single nucleotide variations (SNVs), small to large insertions or deletions (indels), and copy number variations. Furthermore, with higher sensitivity, NGS may identify previously undetected variants. Nevertheless, identifying somatic mutations with small variant allele frequencies (VAFs) requires careful consideration throughout the entire process of acquiring sequencing data, choosing analytic tools, and interpreting final results.

Here, we analyzed peripheral blood samples from patients with unexplained FAP using NGS to estimate the frequency of somatic mosaic mutations in the *APC* gene. We also sought to determine appropriate bioinformatics algorithms for detecting mutations in the *APC* gene with small VAFs in peripheral blood.

## Method

### Patients and samples

Among patients who underwent NGS for hereditary cancer between July 2016 and March 2018, 53 were suspicious for FAP on colonoscopy (Table 1). A list of genes included in the NGS panel is provided in Additional file 1: Table S1. Among these 53 patients, 28 were without detectable pathogenic variants in colonic polyposis associated genes (i.e., *APC, MUTYH, POLE,* and *POLD1*), and they were subjected to further bioinformatics analysis. If available, colonic polyp specimens obtained during colonoscopy were analyzed. Written informed consent was obtained for all patients. The current study was approved by our institutional review board.

**Table 1:** Patients with clinical suspicion of familial adenomatous polyposis and mutation characteristics

Total	53
Phenotype	
Typical FAP	18
Attenuated FAP	35
Age of onset	39 (19–81)
Gene panel results	
Pathogenic *APC* variants	25
Sequence variation	23
Deletion or duplication	2
Patients without pathogenic variants	28
Further analysis	
Mosaicism	7
Unexplained	21

### DNA extraction and sequencing

Genomic DNA was extracted from peripheral blood using the QIAamp DNA Blood Mini Kit (Qiagen, Venlo, The Netherlands). For paraffin-embedded tissue samples, Maxwell® RSC DNA FFPE Kits (Promega, Madison, WI, USA) were used to extract genomic DNA. The amount of input DNA was approximately 500 ng. DNA was fragmented to segments between 150 and 250 bp using the Bioruptor® Pico Sonication System (Diagenode, Liege, Belgium) and then end-repaired and ligated to Illumina adapters (Illumina, San Diego, CA, USA) and indices. Sequencing libraries were then hybridized with capture probes (Celemic, Seoul, Korea). Enriched DNA was then amplified, and clusters were generated and sequenced on a NextSeq 550 instrument (Illumina) with 2 × 151 bp reads. All procedures were performed per the manufacturers’ instructions.

### Data analysis and interpretation

The Burrows-Wheeler alignment tool (0.7.12) was used to align reads to human genomic reference sequences (GRCh37) [13]. To identify SNVs and indels, the HaplotypeCaller in the genome analysis tool kit (GATK) package (3.8–0) was used [14]. All mutations were annotated using ANNOVAR and VEP (87) software [15, 16]. Detected variants were further examined by visual verification using the Integrative Genomic Viewer (IGV) [17]. Variants confirmed to be true-positive were further verified by searching the literature and databases.

In addition to HaplotypeCaller and MuTect2 in GATK (3.8–0) and VarScan2 (2.4.0) were used for further bioinformatics analysis of patients without mutations [18, 19]. To detect medium to large indels, Pindel (0.2.0) was used [20], and results from the four algorithms were compared.

### Confirmation test

Low level variants in two patients were further confirmed using conventional PCR and Sanger sequencing. Two patients with VAFs on below the detection limit of conventional tests were subjected to mutant enrichment with 3′-modified oligonucleotides (MEMO)-PCR, followed by Sanger sequencing, which is based on the use of a 3’modified oligonucleotide primer that blocks extension of the normal allele but enables extension of the mutated allele [21]. Primers used in MEMO–PCR are shown in Additional file 1: Table S2. For another two patients, Sanger sequencing of colonic polyp specimens was performed.

## Results

### Patients and NGS statistics

There were 53 patients with a clinical diagnosis of FAP, and 25 pathogenic variants in *APC* were discovered by NGS for hereditary cancer panel using HaplotypeCaller. Among variants, 23 were sequence variations, and two were partial deletions. They all had VAFs around 0.5 suggestive of germline origin (Additional file 1: Table S3). In the remaining 28 patients, no pathogenic or likely pathogenic variants were observed upon NGS: They comprised patients with a large number of colonic polyps identified on colonoscopy, but no family history of disease associated with colonic polyps (Table 2). One patient had a family history of maternal rectal cancer, although the cancer was not of polyposis type and was diagnosed at the age of 70 years, which can hardly be seen to be associated with *APC* gene mutation. After reanalysis with additional variant calling tools, seven mosaic mutations in *APC* were detected in seven patients, comprising 13.2% (7/53) of all patients suspicious for FAP (Table 2). The median depth of coverage in the gene panel was 691×, with a maximum depth of 7976×. The median depth of coverage for *APC* was 2877×, ranging from 2185× to 4076 × .

**Table 2:** Clinical features and variants detected by NGS in patients with somatic *APC* mosaicism

ID	Age at onset	Number of polyps	Colorectal carcinoma	Family history	Specimen	Mutation detected		Variant callers (variants allele frequency)				Median depth	Confirmation test
								HC	MuTect2	VarScan2	Pindel		
P1	40~49	100 s	No	None	leukocyte	c.3295_3296del	p.Val1099PhefsTer19	ND	0.077	ND	0.068	2668	MEMO-PCR
P1					polyp	c.3295_3296del	p.Val1099PhefsTer19	0.206	0.206	0.228	0.197	790	
P2	40~49	100 s	Adenocarcinoma	None	leukocyte	c.3860_3861dup	p.Gly1288Ter	ND	0.035	0.094	0.032	2497	MEMO-PCR
P3	30~39	200 s	No	None	leukocyte	c.3577_3578del	p.Gln1193ValfsTer14	ND	0.003	ND	0.003	4076	Tissue
P4	50~59	50–70	Adenocarcinoma	None	leukocyte	c.1754delT	p.Leu585ProfsTer5	ND	0.018	ND	0.020	2960	
P5	40~49	30–50	No	Maternal rectal cancer at the age of 70	leukocyte	c.694C > T	p.Arg232Ter	ND	0.034	ND	ND	2185	Tissue
P6	40~49	20–30	No	None	leukocyte	c.3566C > G	p.Ser1189Ter	0.114	0.114	0.114	ND	3624	Sanger sequencing
P7	30~39	300 s	Adenoma	None	leukocyte	c.3211_3238dup	p.Glu1080AlafsTer10	0.195	0.275	ND	0.174	1310	Sanger sequencing

*HC* HaplotypeCaller, *MEMO* Mutant enrichment with 3′-modified oligonucleotides, *ND* Not detected

### Somatic variant detection depends on bioinformatics tools

**Fig. 1:**
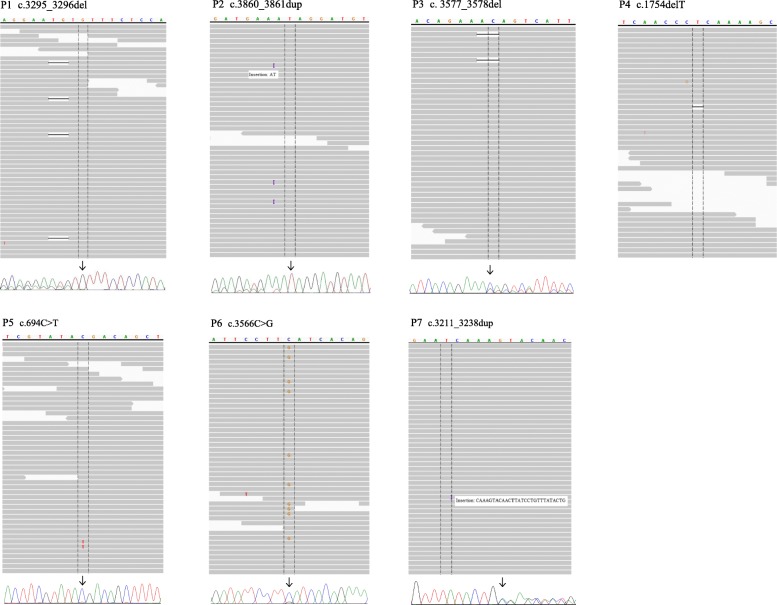
Visual verification of variants with Integrative Genomic Viewer (IGV) and sequencing chromatogram with secondary confirmation test results. Variants with low fractions in IGV reflect NGS results from analyzing peripheral blood. The corresponding sequencing chromatograms are the results of MEMO-PCR of peripheral blood for P1 and P2, conventional PCR of polyp tissue for P3 and P5, and conventional PCR of peripheral blood for P6 and P7

Somatic mosaic mutations detected in *APC* are summarized in Table 2. An additional seven somatic mosaic pathogenic variants were identified by further analysis of sequencing data with MuTect2, VarScan2, and Pindel. The seven mutations are known to cause FAP. Five insertion/deletions resulting in a frameshift mutation were identified by both MuTect2 and Pindel tools, and two nonsense variants went undetected by Pindel, as would be expected. The VAFs thereof range from 0.3 to 7.7%. Only two variants (P2 and P6) with relatively high VAFs were detected by VarScan2, and none of the variants with a VAF below 10% were detected by HaplotypeCaller. All variants were identified by IGV (Fig. 1).

Colonic polyp samples from a patient (P1) were subjected to NGS analysis. As shown in Table 2, a somatic mutation found in leukocytes was enriched in the colonic polyp from 7 to 20%, which confirmed the causative effect of the mutation.

### Confirmation test

Six of the seven somatic variants were further confirmed by a second method (Table 2 and Fig. 1). From patients P1 to P5, variants went undetected by conventional PCR and sequencing using DNA from leukocytes because of low VAF; two variants (P6 and P7) of relatively high VAF were identified by conventional Sanger sequencing. Two pathogenic variants (P1 and P2) were further confirmed using MEMO-PCR, followed by Sanger sequencing. For P3 and P5, colonic polyp specimens were sequenced by conventional PCR and sequencing to confirm the effect of mutations, and suggested that the causative mutations had been enriched and present at higher fractions in polyp tissue.

## Discussion

Familial adenomatous polyposis, an autosomal dominant colorectal tumor syndrome characterized by numerous colorectal adenomatous polyps, is associated with an almost 100% lifetime risk of colorectal cancer if not detected and removed. The majority of patients with FAP harbor a germline mutation in the *APC* gene, and patients typically report family members with the same condition, confirming its autosomal dominant inheritance.

Approximately 10 to 25% of patients with FAP present as sporadic cases [5, 6]. It has been widely recognized that somatic mosaic mutation in *APC* is associated with FAP and is more frequent than previously thought [5, 6, 8, 9]. Since the somatic mutation is invariably a de novo event, patients with mosaic *APC* mutation typically have no family history of FAP. Previous reports have described enrichment of *APC* mutation from white blood cells to colonic mucosa and adenomas, confirming the critical role of mosaic mutation in tumorigenesis [5, 6, 8, 22].

In the present study, seven cases with mosaic *APC* mutations were highly suspected to have FAP based on endoscopic findings, but had no pathogenic variants in genes known to be associated with this condition and no family history of colonic polyposis. The mutation profile of colon tissue was not verified in four patients, and there is a high probability that the colonic lesions shared the same mutation as blood cells. Considering the ectodermal and endodermal origins of blood cells and colonic epithelia, respectively, we presume that the mutations in these cases occurred during early embryogenesis before separation of the two layers [5, 8, 23]. Because this process occurs before germ cell differentiation, the presence of germ cells with the same mutation and transmission thereof to descendants cannot be ruled out. Thus, genetic counseling is necessary, and children of probands might require genetic testing.

*APC* somatic mosaicism is known to be associated with both classical and attenuated FAP [5, 6]. In seven patients with *APC* somatic mosaicism in this study, the median age of onset was 45 years (range 31–53), while that of patients with germline mutations was 34 years. The number of polyps in patients with *APC* somatic mosaicism was round 100 or smaller, while patients with classical FAP presented with more than 100 polyps [24]. Collectively, the patients with somatic mosaic *APC* mutations tended to exhibit an attenuated phenotype.

Testing with NGS and analysis with MuTect2 and Pindel algorithms detected low level mosaic mutations of the *APC* gene that were assumed to cause the disease. While somatic mosaic mutation of the *APC* gene has recently been recognized, conventional sequencing methods have limited sensitivity in the detection thereof. Even with deep sequencing by NGS, variants with low VAF might be missed if analyses are based on the assumption that they are heterozygotes with an allele frequency of at least 0.3. Care must be taken when analyzing and interpreting hereditary cancer genes known to be mutated in a mosaic pattern, such as *APC* and *PPM1D* [25, 26]. The possibility of low-level mosaic mutation should be considered.

There are several previous reports on the detection of somatic mosaic mutations of the *APC* gene [5–11]. To detect low-level mutant alleles, various methods have been used, including denaturing high-performance liquid chromatography, protein truncation test, and high-resolution melting analysis [5–7]. These are less feasible to apply in routine genetic testing for hereditary cancer. We produced sequencing data in a single assay and analyzed them with several algorithms to detect low level variants. GATK HaplotypeCaller is widely used to identify germline variants, and MuTect2 and VarScan2 are optimized to identify variants in cancer specimens [14, 18, 19]. Pindel is a split-read analysis tool for medium to large indels [20]. Among the four variant callers used, only MeTect2 and Pindel could detect low-level mosaic pathogenic variants, with VAFs of 0.2–0.8%. The NGS method has a sensitivity of 10^− 5^~10^− 6^ with adequate sequencing quality and sequencing depth. In addition to adequate analytic tools, it is worth emphasizing the importance of sufficient read depth and careful visual verification to distinguish true variants because tools used to detect low-level variants tend to produce more false positive results.

## Conclusions

We confirmed the clinical utility of NGS testing with adequate combination of bioinformatics tools in detecting low-level somatic variants and deletions in a single assay. We also discovered that mosaic *APC* mutation may be more frequent than previously thought. Accordingly, the presence of mosaic mutation should be considered when analyzing genetic tests in patients with FAP.

## Additional file

Additional file 1: **Table S1.** Genes included in the hereditary cancer panel. **Table S2.** Primers used in the MEMO-PCR to confirm low-level variants in *APC.* **Table S3.** Pathogenic or likely pathogenic germline *APC* variants in patients suspicious for familial adenomatous polyposis. **Table S4.** All variants identified from NGS hereditary cancer panel. (DOCX 58 kb)

## Data Availability

The datasets generated and/or analyzed during the current study are not publicly available due to the policy of the laboratory but are available from the corresponding author on reasonable request.

## Abbreviations

FAP: Familial adenomatous polyposis
GATK: Genome analysis tool kit
IGV: Integrative Genomic Viewer
indels: Insertions or deletions
MEMO: Mutant enrichment with 3′-modified oligonucleotides
NGS: Next-generation sequencing
SNV: Single nucleotide variation
VAF: Variant allele frequency
