# Supplementary material for: Next-generation sequencing with comprehensive bioinformatics analysis facilitates somatic mosaic APC gene mutation detection in patients with familial adenomatous polyposis
Source: BMC Med Genomics. 2019 Jul 3;12:103. doi: 10.1186/s12920-019-0553-0 (PMC6610853; doi:10.1186/s12920-019-0553-0)
Supplement: Supplementary file 1 — Table S1. Genes included in the hereditary cancer panel. Table S2. Primers used in the MEMO-PCR to confirm low-level variants in APC. Table S3. Pathogenic or likely pathogenic germline APC variants in patients suspicious for familial adenomatous polyposis. Table S4. All variants identified from NGS hereditary cancer panel. (DOCX 58 kb) [file 12920_2019_553_MOESM1_ESM.docx]

**Table S1.** Genes included in hereditary cancer panel

APC, ATM, BARD1, BLM, BMPR1A, BRCA1, BRCA2, BRIP1, CDH1, CDK4, CDKN2A, CHEK2, EPCAM, MEN1, MLH1, MRE11A, MSH2, MSH6, MUTYH, NBN, PALB2, PMS2, POLE, PRSS1, PTEN, RAD50, RAD51C, RAD51D, RET, SLX4, SMAD4, STK11, TP53, VHL, WT1, NF1, NF2, RB1, RUNX1, KRAS, NRAS, PTCH1, SDHA, SDHB, ALK, PHOX2B, KIF1B, LMO1, PAX6, CTNNB1, AXIN1, NTRK1, AXIN2, EXO1, FANCM, FLCN, GALNT12, GPC3, GREM1, MLH3, PMS1, POLD1, PPM1D, SDHAF2, RAD51

**Table S2.** Primers used in the MEMO-PCR to confirm low level variants of *APC*

| Patients | Mutation | Primer | Sequence |
| --- | --- | --- | --- |
| APC-1 | p.Val1099PhefsTer19 | Forward | 5’-CCTCAAGTTCCAACCACATTTTGGAC-3’ |
|  |  | Reverse | 5’-CTGCCTCTTGGCATTAGATGAAGGT-3’ |
|  |  | Blocking | 5’-CATTTTGGACAGCAGGAATGTGTTTCTCC-3’ |
| APC-2 | Gly1288Ter | Forward | 5’-TAGTTCATTATCATCTTTGTCATCAGC-3’ |
|  |  | Reverse | 5’-TAAACTAGAACCCTGCAGTCTGCTGG-3’ |
|  |  | Blocking | 5’-CATCAGCTGAAGATGAAATAGGATGTAATC-3’ |

*The 3’-end of the blocking primer was modified with biotin.

**Table S3.** Pathogenic or likely pathogenic germline *APC* variants in patients suspicious for familial adenomatous polyposis

| ID | Age at diagnosis, y | Family history | Exon | DNA change | Affected protein | Variant callers (Variants allele frequency) | | | | Median depth |
| --- | --- | --- | --- | --- | --- | --- | --- | --- | --- | --- |
|  |  |  |  |  |  | HC | MuTect2 | VarScan2 | Pindel |  |
| S185 | 50~59 | none | 4 | c.319dup | p.Ser107PhefsTer32 | 0.54 | 0.53 | 0.61 | 0.33 | 1217 |
| S585 | 20~29 | Paternal colorectal cancer | 16 | c.3631_3632del | p.Met1211ValfsTer5 | 0.50 | 0.50 | 0.55 | 0.48 | 1995 |
| S569 | 30~39 | Maternal colorectal cancer | 16 | c.3183_3187del | p.Gln1062Ter | 0.40 | 0.42 | 0.40 | 0.42 | 1689 |
| S920 | 30~39 | Paternal stomach cancer | 16 | c.4907del | p.Asp1636ValfsTer14 | 0.47 | 0.48 | 0.48 | 0.44 | 1266 |
| S928 | 50~60 | none | 7 | c.694C>T | p.Arg232Ter | 0.48 | 0.48 | 0.48 | ND | 1992 |
| S772 | 20~29 | none | Exon 1 deletion | | |  | | | | |
| S199 | 30~39 | Paternal colorectal cancer | 7 | c.664C>T | p.Gln222Ter | 0.49 | 0.49 | 0.49 | ND | 3961 |
| S465 | 30~39 | Maternal colorectal cancer | 16 | c.3523C>T | p.Gln1175Ter | 0.50 | 0.50 | 0.50 | ND | 2884 |
| S410 | 20~29 | Maternal colorectal cancer | 16 | c.2915del | p.Gly972ValfsTer8 | 0.49 | 0.50 | 0.56 | 0.48 | 2773 |
| S838 | 60~69 | Paternal stomach cancer, Sibling colorectal cancer | 4 | c.288T>A | p.Tyr96Ter | 0.49 | 0.48 | 0.48 | ND | 2692 |
| S696 | 20~29 | Maternal colorectal cancer | 16 | c.2413C>T | p.Arg805Ter | 0.48 | 0.48 | 0.49 | ND | 1516 |
| S961 | 30~39 | Paternal colorectal cancer | 16 | c.2853T>G | p.Tyr951Ter | 0.48 | 0.47 | 0.48 | ND | 1976 |
| S807 | 20~29 | Paternal colorectal cancer | 16 | c.2063C>G | p.Ser688Ter | 0.45 | 0.45 | 0.45 | ND | 1211 |
| S763 | 30~39 | Paternal colorectal cancer | Intron | c.1744-2A>G |  | 0.49 | 0.48 | 0.49 | ND | 691 |
| S302 | 20~29 | none | 16 | c.3833C>G | p.Ser1278Ter | 0.49 | 0.50 | 0.50 | ND | 1120 |
| S420 | 40~49 | none | 16 | c.3927_3931del | p.Glu1309AspfsTer4 | 0.40 | 0.41 | 0.41 | 0.40 | 1198 |
| S169 | 20~29 | none | 16 | c.3927_3931del | p.Glu1309AspfsTer4 | 0.46 | 0.44 | 0.48 | 0.49 | 901 |
| S161 | 20~29 | Paternal colorectal cancer | 16 | c.3927_3931del | p.Glu1309AspfsTer4 | 0.46 | 0.45 | 0.48 | 0.49 | 1028 |
| S857 | 30~39 | none | whole gene deletion | | |  | | | | |
| S872 | 10~19 | none | 16 | c.2626C>T | p.Arg876Ter | 0.47 | 0.47 | 0.48 | ND | 1071 |
| S983 | 50~60 | none | 4 | c.336dup | p.Val113CysfsTer26 | 0.49 | 0.50 | 0.53 | 0.46 | 2810 |
| S803 | 40~49 | Paternal colorectal cancer | 16 | c.2589C>G | p.Tyr863Ter | 0.41 | 0.42 | 0.41 | ND | 1752 |
| S353 | 20~29 | none | 13 | c.1594del | p.Gln532AsnfsTer2 | 0.47 | 0.49 | 0.54 | 0.46 | 522 |
| S887 | 30~39 | none | 16 | c.3183_3187del | p.Gln1062Ter | 0.43 | 0.41 | 0.45 | 0.45 | 2407 |
| S030 | 40~49 | none | 16 | c.3286C>T | p.Gln1096Ter | 0.42 | 0.43 | 0.42 | ND | 1535 |

HC, HaplotypeCaller; ND, not detected

**Table S4.** All variants identified from NGS hereditary cancer panel

| ID | Gene | Accession | DNA change | Affected protein | VAF |
| --- | --- | --- | --- | --- | --- |
| S300 | APC | NM_000038.3 | c.3295_3296del | p.Val1099PhefsTer19 | 0.07 |
| S300 | ATM | NM_000051.3 | c.275A>C | p.Lys92Thr | 0.43 |
| S300 | SLX4 | NM_032444.2 | c.3583_3585del | p.Ile1195del | 0.45 |
| S300 | POLE | NM_006231.2 | c.4290+5C>T |  | 0.50 |
| S409 | APC | NM_000038.5 | c.3860_3861dup | p.Gly1288Ter | 0.03 |
| S409 | FANCM | NM_020937.2 | c.5026G>A | p.Glu1676Lys | 0.47 |
| S409 | AXIN1 | NM_003502.3 | c.879-5T>C |  | 0.43 |
| S286 | APC | NM_000038.5 | c.3577_3578del | p.Gln1193ValfsTer14 | 0.00 |
| S286 | APC | NM_000038.5 | c.5378C>G | p.Ala1793Gly | 0.47 |
| S286 | MUTYH | NM_001128425.1 | c.934-2A>G |  | 0.41 |
| S286 | BRCA2 | NM_000059.3 | c.964A>C | p.Lys322Gln | 0.50 |
| S572 | APC | elNM_000038.3 | c.3294_3295del | p.Leu585ProfsTer5 | 0.02 |
| S572 | PMS2 | NM_000535.5 | c.1659A>G | p.= | 0.49 |
| S502 | APC | NM_000038.5 | c.694C>T | p.Arg232Ter | 0.03 |
| S502 | APC | NM_000038.5 | c.7969G>A | p.Val2657Ile | 0.49 |
| S502 | BRCA2 | NM_000059.3 | c.5252A>G | p.Tyr1751Cys | 0.46 |
| S502 | PRSS1 | NM_002769.4 | c.410C>T | p.Thr137Met | 0.47 |
| S090 | APC | NM_000038.5 | c.3566C>G | p.Ser1189Ter | 0.11 |
| S090 | ATM | NM_000051.3 | c.1607+16A>C |  | 0.52 |
| S090 | GALNT12 | NM_024642.4 | c.719C>T | p.Pro240Leu | 0.49 |
| S090 | SLX4 | NM_032444.2 | c.2235C>T | p.Thr745= | 0.49 |
| S068 | APC | NM_000038.5 | c.3211_3238dup | p.Glu1080AlafsTer10 | 0.17 |
| S068 | CTNNB1 | NM_001904.3 | c.937-10_937-9dup |  | 0.46 |
| S068 | EPCAM | NM_002354.2 | c.298G>A | p.Asp100Asn | 0.50 |
| S185 | APC | NM_000038.5 | c.319dupT | p.Ser107PhefsTer32 | 0.54 |
| S185 | PALB2 | NM_024675.3 | c.2228A>G | p.Tyr743Cys | 0.47 |
| S585 | APC | NM_000038.5 | c.3631_3632del | p.Met1211ValfsTer5 | 0.50 |
| S585 | APC | NM_000038.5 | c.3875C>T | p.Thr1292Met | 0.44 |
| S569 | APC | NM_000038.5 | c.3183_3187del | p.Gln1062Ter | 0.40 |
| S569 | NF1 | NM_001042492.2 | c.4942A>G | p.Thr1648Ala | 0.48 |
| S920 | APC | NM_000038.5 | c.4907del | p.Asp1636ValfsTer14 | 0.47 |
| S920 | POLD1 | NM_002691.3 | c.3065A>G | p.Gln1022Arg | 0.57 |
| S920 | RAD51 | NM_002875.4 | c.645-7C>G |  | 0.45 |
| S928 | APC | NM_000038.5 | c.694C>T | p.Arg232Ter | 0.48 |
| S928 | APC | NM_000038.5 | c.746A>G | p.Lys249Arg | 0.48 |
| S928 | BRIP1 | NM_032043.2 | c.1352C>T | p.Ala451Val | 0.48 |
| S928 | RET | NM_020630.4 | c.874G>A | p.Val292Met | 0.50 |
| S928 | BRCA1 | NM_007294.3 | c.4883T>C | p.Met1628Thr | 0.49 |
| S928 | EPCAM | NM_002354.2 | c.250C>T | p.Pro84Ser | 0.41 |
| S772 | APC | NM_001127511.2 | exon1 deletion |  | 0.59 |
| S772 | EXO1 | NM_130398.3 | c.442C>T | p.Pro148Ser | 0.47 |
| S199 | APC | NM_000038.5 | c.664C>T | p.Gln222Ter | 0.49 |
| S199 | BRIP1 | NM_032043.2 | c.1442G>A | p.Gly481Asp | 0.50 |
| S199 | PHOX2B | NM_003924.3 | c.207C>A | p.= | 0.49 |
| S465 | APC | NM_000038.5 | c.3523C>T | p.Gln1175Ter | 0.50 |
| S465 | RAD51C | NM_058216.2 | c.837+4A>G |  | 0.48 |
| S465 | ATM | NM_000051.3 | c.5369A>G | p.Asp1790Gly | 0.48 |
| S410 | APC | NM_000038.5 | c.2915del | p.Gly972ValfsTer8 | 0.49 |
| S410 | BRCA2 | NM_000059.3 | c.10150C>T | p.Arg3384Ter | 0.46 |
| S410 | SLX4 | NM_032444.2 | c.3583_3585del | p.Ile1195del | 0.41 |
| S838 | APC | NM_000038.5 | c.288T>A | p.Tyr96Ter | 0.49 |
| S838 | NF1 | NM_001128147.2 | c.1740_1742del | p.Phe580del | 0.45 |
| S838 | NF2 | NM_000268 | exon 1 duplication |  | 1.43 |
| S696 | APC | NM_000038.5 | c.2413C>T | p.Arg805Ter | 0.48 |
| S696 | MUTYH | NM_001128425.1 | c.1361A>C | p.Gln454Pro | 0.53 |
| S696 | PTEN | NM_000314.4 | c.-910T>G |  | 0.44 |
| S696 | RAD51 | NM_002875.4 | c.170A>G | p.Lys57Arg | 0.46 |
| S961 | APC | NM_000038.5 | c.2853T>G | p.Tyr951Ter | 0.48 |
| S961 | FANCM | NM_020937.2 | c.4931G>A | p.Arg1644Gln | 0.49 |
| S807 | APC | NM_000038.5 | c.2063C>G | p.Ser688Ter | 0.45 |
| S807 | RUNX1 | NM_001754.4 | c.1415T>C | p.Leu472Pro | 0.49 |
| S807 | PTCH1 | NM_001083603.1 | c.131A>G | p.Glu44Gly | 0.41 |
| S807 | SLX4 | NM_032444.2 | c.2054T>G | p.Val685Gly | 0.45 |
| S807 | APC | NM_000038.5 | c.2298T>C | p.= | 0.43 |
| S807 | POLD1 | NM_001256849.1 | c.909A>G | p.= | 0.28 |
| S807 | POLD1 | NM_001256849.1 | c.957C>T | p.= | 0.77 |
| S763 | APC | NM_000038.5 | c.1744-2A>G |  | 0.49 |
| S302 | APC | NM_000038.5 | c.3833C>G | p.Ser1278Ter | 0.49 |
| S302 | CDKN2A | NM_000077.4 | c.315C>A | p.Asp105Glu | 0.51 |
| S302 | SDHA | NM_004168.2 | c.511C>T | p.Arg171Cys | 0.48 |
| S420 | APC | NM_000038.5 | c.3927_3931del | p.Glu1309AspfsTer4 | 0.40 |
| S420 | APC | NM_000038.5 | c.5257G>C | p.Ala1753Pro | 0.48 |
| S420 | MSH2 | NM_000251.2 | c.1168C>T | p.Leu390Phe | 0.46 |
| S420 | MUTYH | NM_001128425.1 | c.348+11G>A |  | 0.52 |
| S420 | PHOX2B | NM_003924.3 | c.756G>C | p.Ala252= | 0.16 |
| S169 | APC | NM_000038.5 | c.3927_3931del | p.Glu1309AspfsTer4 | 0.46 |
| S169 | PMS2 | NM_000535.5 | c.962T>C | p.Val321Ala | 0.43 |
| S169 | ALK | NM_004304.4 | c.4248G>T | p.Lys1416Asn | 0.46 |
| S161 | APC | NM_000038.5 | c.3927_3931del | p.Glu1309AspfsTer4 | 0.46 |
| S161 | BLM | NM_000057.2 | c.178T>A | p.Leu60Ile | 0.48 |
| S161 | MSH2 | NM_000251.2 | c.1168C>T | p.Leu390Phe | 0.50 |
| S161 | KIF1B | NM_015074.3 | c.1485A>G | p.Pro495= | 0.50 |
| S857 | APC | NM_000038.5 | whole gene deletion |  | 0.58 |
| S872 | APC | NM_000038.5 | c.2626C>T | p.Arg876Ter | 0.47 |
| S872 | MSH2 | NM_000251.2 | c.467A>T | p.Asp156Val | 0.47 |
| S872 | ATM | NM_000051.3 | c.6108T>C | p.Tyr2036= | 0.44 |
| S872 | PTCH1 | NM_000264.3 | c.3338G>A | p.Arg1113His | 0.51 |
| S983 | APC | NM_000038.5 | c.336dup | p.Val113CysfsTer26 | 0.49 |
| S983 | POLE | NM_006231.2 | c.3378+10A>G |  | 0.48 |
| S803 | APC | NM_000038.5 | c.2589C>G | p.Tyr863Ter | 0.41 |
| S803 | KIF1B | NM_015074.3 | c.3649C>T | p.Pro1217Ser | 0.49 |
| S803 | RAD51 | NM_002875.4 | c.1dup | p.Met1? | 0.46 |
| S803 | RAD51 | NM_002875.4 | c.645-7C>G |  | 0.49 |
| S353 | APC | NM_000038.5 | c.1594del | p.Gln532AsnfsTer2 | 0.47 |
| S353 | FLCN | NM_144997.5 | c.1580G>A | p.Arg527Gln | 0.44 |
| S353 | BRCA2 | NM_000059.3 | c.4534C>A | p.Arg1512Ser | 0.44 |
| S353 | AXIN2 | NM_004655.3 | c.2140C>T | p.Arg714Trp | 0.50 |
| S353 | RUNX1 | NM_001754.4 | c.1260C>G | p.Gly420= | 0.05 |
| S353 | RAD51 | NM_002875.4 | c.645-7C>G |  | 0.48 |
| S887 | APC | NM_000038.5 | c.3183_3187del | p.Gln1062Ter | 0.43 |
| S887 | POLE | NM_006231.2 | c.2974G>A | p.Ala992Thr | 0.52 |
| S887 | BRCA2 | NM_000059.3 | c.5969A>C | p.Asp1990Ala | 0.50 |
| S887 | CTNNB1 | NM_001904.3 | c.175A>G | p.Thr59Ala | 0.50 |
| S030 | APC | NM_000038.5 | c.3286C>T | p.Gln1096Ter | 0.42 |
| S268 | NF1 | NM_001128147.2 | c.1740_1742del | p.Phe580del | 0.49 |
| S401 | GALNT12 | NM_024642.4 | c.829G>A | p.Gly277Ser | 0.46 |
| S401 | ALK | NM_004304.4 | c.2210C>T | p.Ser737Leu | 0.48 |
| S401 | STK11 | NM_000455.4 | c.1190C>T | p.Ala397Val | 0.52 |
| S401 | MSH2 | NM_000251.2 | c.1886A>G | p.Gln629Arg | 0.49 |
| S401 | RET | NM_020630.4 | c.833C>A | p.Thr278Asn | 0.50 |
| S401 | NBN | NM_002485.4 | c.1657A>G | p.Met553Val | 0.49 |
| S424 | MSH6 | NM_001281492.1 | c.383T>C | p.Ile128Thr | 0.44 |
| S171 | APC | NM_000038.5 | c.6896C>T | p.Pro2299Leu | 0.53 |
| S171 | RET | NM_020630.4 | c.1759+10G>T |  | 0.47 |
| S171 | RAD50 | NM_005732.3 | c.699G>A | p.= | 0.49 |
| S558 | APC | NM_001127510.2 | c.423-8A>G |  | 0.53 |
| S558 | BRCA1 | NM_007294.3 | c.3649T>C | p.Ser1217Pro | 0.48 |
| S558 | BARD1 | NM_000465.2 | c.30_44del | p.Gln11_Arg15del | 0.32 |
| S558 | BRIP1 | NM_032043.2 | c.1442G>A | p.Gly481Asp | 0.49 |
| S558 | SLX4 | NM_032444.2 | c.2235C>T | p.= | 0.58 |
| S558 | MSH2 | NM_000251.2 | c.942+27_942+29del |  | 0.44 |
| S333 | BRIP1 | NM_032043.2 | c.2554A>G | p.Asn852Asp | 0.45 |
| S333 | MLH3 | NM_001040108.1 | c.3488G>A | p.Gly1163Asp | 0.43 |
| S304 | AXIN1 | NM_003502.3 | c.853C>T | p.Arg285Trp | 0.56 |
| S304 | KRAS | NM_004985.3 | c.556G>A | p.Val186Ile | 0.40 |
| S304 | NTRK1 | NM_001012331.1 | c.97G>T | p.Ala33Ser | 0.52 |
| S959 | FANCM | NM_020937.2 | c.4931G>A | p.Arg1644Gln | 0.51 |
| S959 | BLM | NM_000057.2 | c.1785A>T | p.= | 0.49 |
| S476 | MSH6 | NM_000179.2 | c.4068_4071dup | p.Lys1358AspfsTer2 | 0.47 |
| S476 | POLE | NM_006231.2 | c.6135C>T | p.= | 0.45 |
| S671 | PMS1 | NM_000534.4 | c.1258del | p.His420IlefsTer22 | 0.49 |
| S671 | BRCA2 | NM_000059.3 | c.7706G>A | p.Gly2569Asp | 0.49 |
| S671 | AXIN2 | NM_004655.3 | c.426C>T | p.= | 0.48 |
| S671 | FANCM | NM_020937.2 | c.2240A>G | p.His747Arg | 0.50 |
| S929 | FLCN | NM_144997.5 | c.205G>A | p.Val69Ile | 0.49 |
| S929 | ALK | NM_004304.4 | c.487G>T | p.Val163Leu | 0.50 |
| S334 | None |  |  |  |  |
| S253 | SLX4 | NM_032444.2 | c.4057C>T | p.His1353Tyr | 0.54 |
| S253 | NF1 | NM_001128147.2 | c.1740_1742del | p.Phe580del | 0.41 |
| S253 | PHOX2B | NM_003924.3 | c.765_779del | p.Ala256_Ala260del | 0.48 |
| S253 | POLE | NM_006231.2 | c.4290+5C>T |  | 0.43 |
| S828 | BMPR1A | NM_004329.2 | c.1243G>A | p.Glu415Lys | 0.43 |
| S828 | BLM | NM_000057.2 | c.2839A>G | p.Ile947Val | 0.64 |
| S828 | NF1 | NM_001042492.2 | c.5160G>T | p.Glu1720Asp | 0.45 |
| S828 | NF2 | NM_016418.5 | c.240+15C>T |  | 0.52 |
| S865 | BRCA2 | NM_000059.3 | c.4320A>C | p.Lys1440Asn | 0.50 |
| S396 | APC | NM_000038.5 | c.1276G>T | p.Ala426Ser | 0.45 |
| S396 | STK11 | NM_000455.4 | c.842C>T | p.Pro281Leu | 0.49 |
| S396 | PTCH1 | NM_001083603.1 | c.86G>T | p.Gly29Val | 0.55 |
| S899 | MUTYH | NM_001128425.1 | c.857G>A | p.Gly286Glu | 0.46 |
| S899 | MUTYH | NM_001128425.1 | c.842C>T | p.Ala281Val | 0.53 |
| S899 | CDH1 | NM_004360.3 | c.2494G>A | p.Val832Met | 0.48 |
| S899 | PTCH1 | NM_001083603.1 | c.86G>T | p.Gly29Val | 0.43 |
| S118 | APC | NM_000038.5 | c.1276G>T | p.Ala426Ser | 0.49 |
| S118 | PRSS1 | NM_002769.4 | c.107C>G | p.Pro36Arg | 0.43 |
| S118 | BLM | NM_000057.2 | c.178T>A | p.Leu60Ile | 0.50 |
| S118 | WT1 | NM_024426.4 | c.1433-10G>A |  | 0.51 |
| S230 | APC | NM_000038.5 | c.7150T>A | p.Leu2384Ile | 0.50 |
| S791 | MSH6 | NM_000179.2 | c.4068_4071dup | p.Lys1358AspfsTer2 | 0.49 |
| S791 | SDHB | NM_003000.2 | c.541-3C>T |  | 0.51 |
| S791 | PTCH1 | NM_000264.3 | c.3964G>A | p.Ala1322Thr | 0.36 |
| S791 | FANCM | NM_020937.2 | c.925G>A | p.Glu309Lys | 0.52 |
| S485 | MSH2 | NM_000251.2 | c.1168C>T | p.Leu390Phe | 0.51 |

VAF, variant allele frequency
